# Sodium-Glucose Cotransporter-2 Inhibitors in Diabetic Patients with Heart Failure: An Update

**DOI:** 10.3390/ph17111419

**Published:** 2024-10-23

**Authors:** Nicia I. Profili, Roberto Castelli, Antonio Gidaro, Roberto Manetti, Margherita Maioli, Alessandro P. Delitala

**Affiliations:** 1 Department of Medicine, Surgery and Pharmacy, University of Sassari, 07100 Sassari, Italyrmanetti@uniss.it (R.M.); 2 Department of Biomedical and Clinical Sciences Luigi Sacco, Luigi Sacco Hospital, University of Milan, 20157 Milan, Italy; gidaro.antonio@asst-fbf-sacco.it; 3 Department of Biochemical Science, University of Sassari, 07100 Sassari, Italy; mmaioli@uniss.it

**Keywords:** SGLT2i, Na^+^-glucose cotransporter-2 inhibitors, chronic heart failure, acute heart failure, diabetes, cardiovascular disease

## Abstract

Diabetes mellitus and heart failure are two diseases that are commonly found together, in particular in older patients. High blood glucose has a detrimental effect on the cardiovascular system, and worse glycemic control contributes to the onset and the recrudesce of heart failure. Therefore, any specific treatment aimed to reduce glycated hemoglobin may, in turn, have a beneficial effect on heart failure. Sodium-glucose cotransporter-2 inhibitors have been initially developed for the treatment of type 2 diabetes mellitus, and their significant action is to increase glycosuria, which in turn causes a reduction in glucose blood level and contributes to the reduction of cardiovascular risk. However, recent clinical trials have progressively demonstrated that the glycosuric effect of the sodium-glucose cotransporter-2 inhibitors also have a diuretic effect, which is a crucial target in the management of patients with heart failure. Additional studies also documented that sodium-glucose cotransporter-2 inhibitors improve the therapeutical management of heart failure, independently by the glycemic control and, therefore, by the presence of diabetes mellitus. In this review, we analyzed studies and trials demonstrating the efficacy of sodium-glucose cotransporter-2 inhibitors in treating chronic and acute heart failure.

## 1. Introduction

Diabetes is a heterogeneous disease with an increasing prevalence worldwide. Some regions of the world are experiencing rapid growth in frequency, particularly in eastern countries [1]. The causes of this epidemiological trend can be mainly found in unhealthy obesogenic diets and reduced physical activity. Patients with diabetes have an increased risk of developing cardiovascular disease (CVD): heart failure (HF), coronary artery disease, atrial fibrillation, and stroke. Diabetic patients are also at increased risk of developing chronic kidney disease (CKD) and have a higher risk of all-cause mortality, mainly due to the combination of cardiovascular and kidney disease. The treatment of type 2 diabetes has been profoundly changed in the last years due to the possibility of prescribing the inhibitors of Na+-glucose cotransporter-2 (SGLT2i), which has a good effect on glycated hemoglobin reduction. Further studies also demonstrated a broader impact, not limited to diabetes. Indeed, due to its glycosuric action, it has been shown that SGLT2i could also be used for the treatment of HF as well as CKD. In this narrative review, we focused on the cardiac effect of this drug, analyzing the central studies that demonstrated the positive impact that SGLT2i has on the management of HF.

## 2. Mechanism of Action of Sodium-Glucose Cotransporter-2 Inhibitors

The kidney plays a critical role in glucose homeostasis. Indeed, it contributes to gluconeogenesis (15–55 g/die) and reabsorbs the glucose filtered into the glomerular filtrate. Glycosuria occurs when blood glucose exceeds 180 mg/dL, thus usually reabsorbing all the daily glucose filtered in healthy individuals [2]. Na^+^-glucose cotransporter-2 (SGLT2) is the principal cotransporter responsible for the reabsorption of filtered glucose and is expressed in the luminal membrane in the early portion of the kidney’s proximal tubule, where 80–90% of the filtered glucose is physiologically absorbed [3]. The remaining 10–20% is adsorbed by Na^+^-glucose cotransporter-1 (SGLT1) in the distal segment of the renal proximal tubule. SGTL2 has a high capacity and low affinity for glucose transport, which occurs against a concentration gradient. Further, its transport is coupled with the downhill of Na^+^, which is then actively extruded in the basolateral surface of the cell. Glucose transporter 2 (GLUT2) carries glucose in the blood by facilitated diffusion [3]. The proximal tubule can increase the glucose reabsorption along with the rising of plasma glucose level until the transport maximum for glucose is reached, which is usually set at 260–350 mg/min. Once past this threshold, which is roughly equal to 180–200 mg/dL of blood glucose, the SGTL capacity is saturated, and glucose begins to be excreted via the urine. The blood glucose threshold has been demonstrated to be higher in diabetic patients (e.g., 220 mg/dL).

Inhibition of SGLT2 can thus increase glucose excretion in the urine by lowering the renal threshold for glucose excretion. Indeed, SGLT2i reduces the reabsorption of 30–50% of the glucose filtered by the kidney. This action is independent from insulin [2].

## 3. Heart Failure

Cardiovascular disease is the leading cause of morbidity and mortality, and different causes may contribute to its development [4,5].

HF is a clinical syndrome characterized by specific symptoms (breathlessness, ankle swelling, and fatigue) and signs (pulmonary crackles, peripheral edema, and elevated jugular venous pressure). Several conditions may lead to HF, and its diagnosis—and specific treatment—is mandatory. Incidence of HF is dramatically increasing worldwide: in Europe, it is about 5/1000 person-years in adults [6], with an apparent increase with age (>10% in subjects aged over 70). But the real-world prevalence is likely higher than those reported in the studies that included diagnosed HF [7].

Traditionally, HF is divided into three different phenotypes related to the value of left ventricular ejection fraction (LVEF) [6]. Reduced LVEF is defined as ≤40% and designated as HFrEF, while patients with LVEF between 41% and 49% had mildly reduced left ventricular function (NFmrEF). SGLT2i with preserved ejection fraction (HFpEF) is defined as the presence of clinical diagnosis of HF without evidence of structural and/or functional cardiac abnormalities and/or raised natriuretic peptides and with LVEF ≥ 50%.

## 4. SGLT2i in Heart Failure: Mechanisms of Action

**Figure 1:**
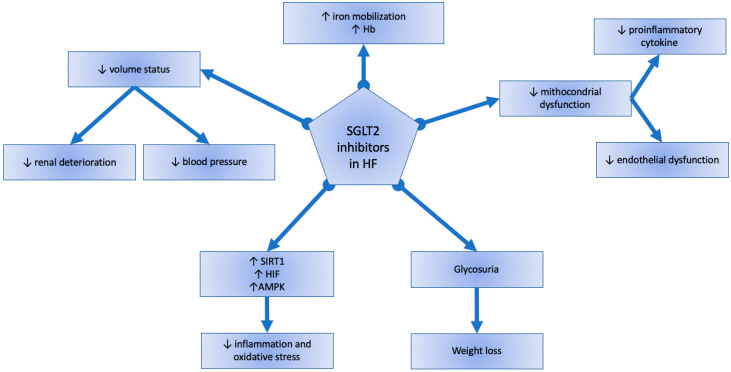
Mechanisms of SGLT2 inhibitors in heart failure.

SGLT2i exhibits pleiotropic effects on different physiological systems, some of which are independent of the anti-hyperglycemic effect (Figure 1). The most plausible hypothesis is the augmented diuretic effect secondary to glucosuria and natriuresis. The effect starts within 24 h, which leads to a 300 mL/day increase in urinary output [6] and decreases after 12 weeks of treatment [8]. Studies also reported that the diuretic effect is more efficient when SGTL2 inhibitors are associated with loop diuretics [9], although other reports argue against the possible diuretic effect. Indeed, the EMPA-RESPONSE-AHF study reported that Empagliflozin did not reduce dyspnea scores [10]. Further, another study showed that Dapagliflozin was not associated with an acute drop in NT-proBNP in patients with SGLT2i with reduced ejection fraction [11]. Studies that supported the possible diuretic effect also reported to low blood pressure and reduced renal deterioration. Indeed, studies have reported a reduction of intraglomerular hypertension, modulation of the sympathetic nervous system, and reduction of oxidative stress and inflammation [12]. However, it should be noted that the effect of SGTL2i on the cardiorenal axis was higher in hospitalized patients with HFrEF than in those with HFpEF [13]. SGTL2i also had a beneficial effect on the cardiomyocytes of mouse models that developed a reduction of cardiac fibrosis through different pathways: reduced expression of fibronectin 1, collage type I and III, and transforming growth factor-β [14]. Another critical mechanism of SGLT2i is the reduced oxidative stress and inflammation, as showed by some studies which reported a reduction of circulating pro-inflammatory factors (C-reactive protein, interleukin-6, and tumor necrosis factor-α) [15] and is mostly multifactorial. In addition, SGLT2i inhibits the NLRP3 inflammasome, which has a clear role in chronic inflammation in some CVD. This effect leads to decreased macrophage infiltration and favours the release of specific cytokine [16]. Lastly, some studies reported an increased autophagic flux, which is a measure of autophagic degradation activity. Patients with HF had an impaired autophagy, which physiologically contributes to the degradation of dysfunctional mitochondria, thus reducing the oxidative stress [17]. Recent studies showed that SGLT2i can induce autophagy, mainly through an increase of AMPK, sirtuins, and HIF, and a reduction of mTOR [18].

## 5. SGLT2i and Chronic Heart Failure with Reduced Left Ventricular Ejection Fraction

The Dapagliflozin and Prevention of Adverse Outcomes in SGLT2i (DAPA-HF) trial was the first study that tested the efficacy of Dapagliflozin in patients with chronic HFrEF [19], as reported in Table 1. This study randomly assigned 4744 patients with HFrEF to receive either 10 mg/day Dapagliflozin or placebo in addition to recommended therapy, aiming to evaluate, as a primary outcome, a composite of worsening SGLT2i or cardiovascular death. At the end of the follow-up period (median 18.2 months), the authors found a lower frequency of primary outcome in patients treated with SGTL2 inhibitors compared to placebo (16.3% vs. 21.2%, HR 0.74; 95%CI 0.65–0.85). Similarly, the frequency of death from cardiovascular causes was lower in patients treated with Dapagliflozin (9.6% vs. 11.5%, HR 0.82; 95%CI 0.69–0.98). Interestingly, the main findings were comparable between patients with diabetes and those without diabetes. In addition, the frequency of adverse effects was similar between the groups. Post hoc analyses from the same trial also revealed that SGLT2i improved specific clinical outcomes regardless of frailty [20], race [21], and atrial fibrillation [22].

Similarly, the EMPEROR-Reduced trial is another randomized, double-blind, parallel-group, placebo-controlled trial that focused on patients with chronic HF (functional class II, III, or IV) with a left ventricular ejection fraction of 40% or less [23]. The sample consisted of 3730 patients who received either Empagliflozin 10 mg/daily or placebo and were followed for a median of 16 months. The risk of composite outcome (cardiovascular death or hospitalization for worsening SGLT2i) was reduced in patients who received SGLT2i compared to placebo (HR 0.75, 95%CI 0.58–0.85, *p* < 0.001) regardless of the presence of diabetes. Again, post hoc analyses of this trial revealed additional positive effects: reduced worsening of SGLT2i even in outpatients, with benefits seen early after initiation [24].

Another multicenter, randomized, double-blind clinical trial enrolled 90 patients with HFrEF, randomly assigned to receive either Dapagliflozin or placebo [25]. Follow-up was set at 1 and 3 months to evaluate changes in maximal functional capacity. The authors found a significant improvement in peak VO2 at 1 and 3 months, thus resulting in an early improvement in maximal exercise capacity. Finally, the DEFINE-HF trial demonstrated that patients treated with Dapagliflozin experienced an improvement in lung fluid volumes [26].

**Table 1:** Studies that tested SGLT2i in chronic heart failure with reduced left ventricular ejection fraction.

Study	*n*	Diabetes	SGTL2i	Follow Up	Outcomes	Events P.O.		Result
						SGTL2i	Placebo	
DAPA-HF [19]	4744	45.0%	D	18.2 months	Composite outcome of worsening SGLT2i or death from CV causes	386	502	Reduced risk
					Composite of hospitalization for HF or CV death	382	495	Reduced risk
					Composite of number of hospitalizations for HF and CV death	567	742	Reduced risk
					Composite of worsening renal function (decline in the eGFR or renal death)	28	39	No effect
					Death from any cause	276	329	No effect
				8 months	Change from baseline of KCCQ	N/A	N/A	Improved patient-reported symptoms
DAPA-HF [20]	4742			18.2 months	Worsening of HF or CV death accordingly to the frailty index	N/A	N/A	Reduced risk regardless of frailty status. Absolute reductions were larger in more frail patients.
Palau et al. [25]	90	54.5%	D	1 and 3 months	Change from baseline in mean peakVO2	N/A	N/A	Improvement in peakVO2 at 1 and 3 months
EMPIRE HF [27]	190	20.0%	E	90 days	Change of N-terminal pro-brain natriuretic peptide (NT-proBNP)	N/A	N/A	No change
EMPIRE HF [28]	190	20.0%	E	12 weeks	Changes in erythropoiesis and iron metabolism	N/A	N/A	Increased erythropoiesis and augmented early iron utilization
EMPEROR-Reduced 32865377	3730	49.8%	E	18 months	Composite of cardiovascular death or hospitalization for worsening SGLT2i	361	462	Reduced risk
EMPEROR-Reduced [29]	3730	49.8%	E	12, 32, and 52 weeks	Changes in body weight	N/A	N/A	Benefits of SGTL2i were present across all BMI categories. Weight loss was associated with higher risk of all-cause mortality, regardless of treatment group.
DEFINE_HF [26]	85	75.6%	D	12 weeks	Changes in lung fluid volumes	N/A	N/A	Reduced lung congestion

Abbreviations: D, Dapagliflozin; E, Empagliflozin; heart failure, HF; CV, cardiovascular; N/A, not applicable; KCCQ-CS, Kansas City Cardiomyopathy Questionnaire; eGFR, estimated glomerular filtrate rate; SGLT2i, Na^+^-glucose cotransporter-2 inhibitorsSGLT2i and chronic heart failure with mildly reduced left ventricular ejection fraction.

The DELIVER trial was a double-blind, randomized, controlled study that tested the efficacy of Dapagliflozin in patients with NFmrEF or HFrEF, as reported in Table 2. The study randomly assigned 6263 patients to Dapagliflozin 10 mg/day or placebo in addition to usual therapy to test the primary outcome (composite of worsening of HF-defined as unplanned hospitalization or urgent visit for HF or cardiovascular death) [30]. Results of the analyses revealed that worsening of SGLT2i occurred less frequently in patients treated with SGTL2 inhibitors (11.8% vs. 14.5%, HR 0.79, 95%CI, 0.69–0.91) as well as cardiovascular death (7.4% vs. 8.3%, HR 0.88, 95%CI 0.74–1.05). The presence of NFmrEF or HFpEF did not affect the results, as well as the presence of diabetes. The incidence of adverse effects was comparable between the two groups. The same trial also found patients treated with Dapagliflozin had a mild decline in estimated glomerular filtration rate, which was not associated with subsequent risk of cardiovascular event of acute kidney injury [31]. Further, chronic obstructive pulmonary disease, which is common in patients with HF, did not affect the beneficial effect of Dapagliflozin [32].

## 6. SGLT2i and Chronic Heart Failure with Preserved Left Ventricular Ejection Fraction

HFpEF accounts for at least half of the patients with SGLT2i. Clinical trials for HFpEF gave different results. Indeed, while some previous studies reported no positive effect on mortality and limited impact on HF hospitalizations, recent guidelines suggested the prescription of SGTL2 inhibitors for the management of HF. The EMPERIAL-Preserved Trial tested the effect of Empagliflozin on exercise ability and HF symptoms [33]. The authors enrolled HFpEF and HFrEF, with or without type 2 diabetes mellitus, and treated with Empagliflozin 10 mg/day or placebo for 12 weeks to assess a change in the 6-min walk test distance. The authors also evaluated symptoms of SGLT2i through the Kansas City Cardiomyopathy Questionnaire Total Symptom Score (KCCQ-TSS) and Chronic SGLT2i Questionnaire Self-Administered Standardized format (CHQ-SAS) dyspnea score. Analyses showed no effect on exercise ability or specific dyspnea score in both types of HF. This study was somewhat limited by the small sample size and by the short follow-up, which did not allow the evaluation of specific outcomes (mortality and hospitalization). The EMPEROR-Preserved trial was a multicenter, double-blinded, placebo-controlled randomized trial that assessed Empagliflozin’s effects on a composite of cardiovascular death or hospitalization for SGLT2i [34]. The study included 5988 patients with HFpEF (defined as ejection fraction > 40%), who were treated with Empagliflozin 10 mg/daily or placebo in addition to the usual therapy for a median of 26.2 months. Primary outcomes showed a 21% reduction in patients treated with SGTL2 inhibitors, mainly driven by the reduction of hospitalization for SGLT2i (HR 0.79%; 95%CI 0.69–0.90, *p* < 0.001). Indeed, the incidence of cardiovascular death was lower but not significant (HR 0.91, 95%CI 0.76–1.0). This trial also showed a benefit of the use of Empagliflozin in the secondary outcomes: reduction of eGFR decline and total SGLT2i hospitalization, and a modest improvement in quality of life, regardless of the presence of diabetes. Another trial, PRESERVED-HF, focused on the effects of SGTL2 inhibitors on symptoms and exercise function in HFpEF patients [35]. The study, which was a multicenter, double-blinded, placebo-controlled study, included 324 patients treated either with Empagliflozin 10 mg/daily or placebo. At 12 weeks, using SGTL2 inhibitor improved the Kansas City Cardiomyopathy Questionnaire Clinical Summary Score (KCCQ-CS) and weight, natriuretic peptides, glycated hemoglobin, and systolic blood pressure. The benefit of Empagliflozin on HFpEF patients was independent of diabetes.

The results of the DELIVER trial were in line with previous studies. This multicenter, double-blind, placebo-controlled, randomized study assessed whether Dapagliflozin 10 mg/daily would improve worsening SGLT2i or cardiovascular death (primary composite outcome) in symptomatic stable HFpEF patients, with or without diabetes mellitus [30]. The study enrolled 6263 patients randomized to either Dapagliflozin 10 mg/daily or placebo in addition to the usual therapy. Over a median of 2.3 years, the primary outcome occurred less frequently in the Dapagliflozin group (16.4% vs. 19.5%, HR 0.73, 95%CI 0.73–0.92, *p* < 0.001), mainly driven by the reduction of worsening of SGLT2i (11.8% vs. 14.5%, HR 0.79, 95%CI 0.69–0.91) as compared to cardiovascular death (7.4% vs. 8.3%, HR 0.88, 95%CI 0.74–1.05).

## 7. SGLT2i and Acute Heart Failure

Studies tested the benefit of SGTL2 inhibitors for treating acute decompensated SGLT2i in hospitalized patients (Table 3). The EMPULSE trial analyzed 530 patients hospitalized for decompensated SGLT2i irrespective of left ventricular ejection fraction [36]. Compared to placebo, subjects treated with Empagliflozin 10 mg daily within 5 days of admission had a significant clinical benefit, defined as a hierarchical composite of death from any cause, number of SGLT2i events, and Kansas City Cardiomyopathy Questionnaire total symptom score. The EMPAG-HF focused on cumulative urine output over 5 days [37]. This single-center prospective, double-blind, placebo-controlled study randomized 59 patients within 12 h of hospitalization for acute decompensated HF. Patients, in addition to the standard decongestive treatments, were randomly assigned to Empagliflozin 25 mg daily or placebo. The authors reported a 25% increase in cumulative urine output without a decline of glomerular filtration rate. Similar results were obtained in the EMPA-RESPONSE-AHF, which demonstrated the beneficial effect of Empagliflozin on patients treated within 24 h of the presentation to the hospital [10]. Albeit the authors did not find changes in Visual Analogue Scale dyspnea, diuretic response, and length of hospital stay, they reported an increased urinary output and reduced combined endpoint (worsening SGLT2i, hospitalization for SGLT2i, or death at 60 days). It should be noted that all these studies tested SGTL2 inhibitors during low doses of intravenous furosemide. The DAPA-RESIST study tested the effect of SGTL2 inhibitors in patients with diuretic resistance, defined as insufficient decongestion despite treatment, with a high dose of intravenous furosemide (≥160 mg/day) [38]. Patients were randomized to Dapagliflozin 10 mg/day or Metolazone 5–10 mg/day for a 3-day treatment period. The authors found that patients treated with Dapagliflozin received a larger cumulative dose of furosemide but without a more efficient relief of pulmonary congestion. A significant weight reduction at up to 96 h of Dapagliflozin was also documented.

Recent studies also focused on early prescriptions of SGTL2 inhibitors. The SOLOIST-WHF, a multicenter, double-blind trial, randomized 608 patients to Sotagliflozin and 614 to placebo and administered before discharge (48.8%) and a median of 2 days after discharge (51.2%). The primary endpoint was death from cardiovascular causes and hospitalization or urgent visits for SGLT2i [39]. Patients were followed for a median of 9 months, and 600 primary endpoints occurred. The rate of cardiovascular death was lower in the Sotagliflozin group (HR 0.84; 95%CI 0.58–1.22), while the frequency of acute kidney injury was similar to those who had a placebo. A post hoc analysis also demonstrated that starting Sotagliflozin before the discharge significantly decreased cardiovascular death and SGLT2i events 30 and 90 days after the discharge [40]. Another retrospective analysis by Burgos et al. pointed out that in-hospital initiation of SGLT-2 inhibitors was associated with significantly higher prescription rates and lower prevalence of hospitalization or urgent visits for acute SGLT2i or all-cause mortality at 90 days [41].

Current data suggest a role for SGTL2 inhibitors in the treatment of acute decompensated SGLT2i. However, they must be considered an additional therapy that cannot replace the loop diuretic, which is the landmark for the treatment of acute SGLT2i. Studies also suggest starting SGLT2 early, which is substantially equivalent when administered within 12 h of the onset of acute SGLT2i or the days before discharge from the hospital.

## 8. Additional Effects of SGTL2 Inhibitors

### 8.1. SGLT2i and Left Ventricular Mass

The EMPA-HEART CardioLink-6 study evaluated the effect of Empagliflozin on left ventricular mass in patients with coronary artery disease and type 2 diabetes mellitus. The authors recruited 97 subjects randomized to Empagliflozin 10 mg/day or placebo. The primary outcome was the 6-month change in left ventricular mass indexed to the body surface assessed by cardiac resonance imaging. Authors reported a regression of mean left ventricular mass in patients treated with SGTL2i compared to placebo (−2.6 g/m^2^ vs. 0.01 g/m^2^, *p* = 0.01). Further, the Empagliflozin group had a reduction of systolic and diastolic blood pressure (respectively, −6.8 mmHg vs. −2.3 mmHg *p* = 0.003 and −3.2 mmHg vs. −0.6 mmHg *p* = 0.02) and an elevation of hematocrit (*p* = 0.0003) [42]. To rule out the anti-hyperglycemic effect, the EMPA-TROPISM study evaluated the left ventricular mass on 84 nondiabetic patients with SGLT2i with reduced ejection fraction, which were randomized to Empagliflozin 10 mg/daily or placebo. After 6 months, these patients showed a significant decrease in left ventricular mass, other than an improvement in ejection fraction and 6-min walk [43].

### 8.2. SGLT2i and Acid Uric Metabolism

The association between hyperuricemia and HF is well acknowledged. Serum uric acid is an oxidative stress index that contributes to endothelial dysfunction by impairing nitric oxide production, considered a prognostic index in patients with preexisting HF [44]. The exact pathophysiological link between the two diseases is not clear, but it has been demonstrated that serum acid uric concentration is related to greater activity of superoxide dismutase and endothelium-dependent vasodilatation [40], and other studies showed a possible further link with inflammation. Indeed, hyperuricemia is associated with interleukin-6, neutrophil count, and C-reactive protein, all specific markers of proinflammatory state associated with an increased risk of HF [44,45,46]. In addition, hyperuricemia has been associated with an increased risk of developing hypertension and coronary heart disease [47,48], which can further explain the link between increased uric acid and HF.

SGTL2i act on different pathways of uric acid metabolism. Indeed, SGTL2i decrease purine synthesis, downregulate different enzymes of the pentose phosphate pathways, and reduce intracellular levels of hypoxanthine, reducing NADPH oxidase activity [49,50]. The effect of SGTL2i is not limited to lowering uric acid synthesis but also promotes its excretion. Indeed, these drugs show a uricosuric effect, which is strictly connected to the glycosuric effect. Indeed, SGTL2i may upregulate ABCG2 and can downregulate URAT1, which is a major protein involved in uric acid reabsorption [51,52].

### 8.3. SGLT2i and Iron Metabolism

Absolute or relative iron deficiency is commonly found in patients with HF. Reduced dietary intake, chronic blood loss, and impaired absorption, which can be secondary to gut edema, the use of specific drugs, and/or chronic inflammation, are all factors that can contribute to iron deficiency. Anemia is less frequent but recognizes the same causes [53]. It is well acknowledged that iron deficiency has a worse impact in patients with HF, and several randomized trials have documented an improvement after treatment with ferric Carboxymaltose [54]. An increased hematocrit and hemoglobin are frequently found in HF patients treated with SGLT2i, but it is not clear whether this effect is secondary to hemoconcentration or increased erythropoiesis. The DAPA-HF examined iron deficiency’s prevalence and consequences in patients with HF treated with SGLT2i [55]. The study found that 43.7% of the sample had iron deficiency, with an increased rate of worsening SGLT2i (hospitalization or urgent visit requiring intravenous treatment) compared to those with normal blood iron. Analyses showed a trend for the beneficial effect of Dapagliflozin on worsening SGLT2i was greater in patients with iron deficiency compared to those iron-repleted (HR 0.74 95%CI 0.58–0.92 vs. HR 0.81, 95%CI 0.63–1.03, p-interaction = 0.59). In addition, the authors also found that patients treated with Dapagliflozin also had a reduction of transferrin saturation, ferritin, and hepcidin. In contrast, iron-binding capacity and soluble transferrin receptors increased compared to the placebo. Therefore, the authors pointed out that Dapagliflozin increased iron use and, at the same time, improved clinical outcomes regardless of baseline iron status. Another trial aimed to investigate the early effect of Empagliflozin on iron metabolism and erythropoiesis in patients with HFrEF [28]. Patients were randomly assigned to either Empagliflozin or placebo for 12 weeks, and the analyses suggested that the use of SGLT2i increased erythropoiesis and augmented early iron utilization, contributing to a cardioprotective effect. This result is somewhat consistent with findings reported by Docherty et al. [56]. The study was a post hoc exploratory analysis of the IRONMAN trial, which randomized patients with SGLT2i with iron deficiency to intravenous ferric Derisomaltose or usual care. Analyses reported a trend of a more significant increase in hemoglobin in patients treated with ferric Derisomaltose, which had SGLT2i at baseline.

Overall, it is still not clear whether anemia simply reflects a marker of poor prognosis in patients with HFrEF or is a therapeutic goal that needs to be treated. Indeed, while some studies reported Dapagliflozin corrected anemia more frequently than placebo, thus improving outcomes, other studies failed to demonstrate improvement in cardiovascular outcomes in anemic patients with SGLT2i treated with Darbepoetin alfa [57]. Therefore, it is still to be elucidated whether SGLT2i has a synergistic effect with iron therapy in some patients with HFrEF.

## 9. Conclusions

SGLT2i have dramatically improved the management of diabetes, and their use for the treatment of HF is now strengthened by the growing evidence that suggested beneficial effects in almost all types of HF. The mechanisms underlying the cardioprotective effect of SGLT2i are multiple: lowering blood pressure, enhancing diuresis, improving glycemic control, and preventing inflammation. Possible adverse effects may limit their prescription in selected patients, in particular in those who experienced genital mycotic infection, pyelonephritis, and acute kidney injury. Evidence suggests that all these mechanisms collectively contribute to the benefits documented by the different clinical trials in patients with HF.

## Figures and Tables

**Table 2:** Studies that tested SGLT2i in chronic heart failure with mildly reduced left ventricular ejection fraction and preserved left ventricular ejection fraction.

Study	*n*		Type of HF	Diabetes	SGTL2i	Follow Up	Outcomes	Events P.O.		Result
								SGTL2i	Placebo	
DELIVER [31]	5788		NFmrEF HFpEF	42–50%	D	1 month	Kidney composite outcome (first occurrence of ≥50% decline in eGFR within 1 month, development of end-stage kidney disease, or death to kidney cause)	N/A	N/A	Initial eGFR decline after Dapagliflozin, which was not associated with subsequent risk of cardiovascular or kidney events.
DELIVER [30]	2216		NFmrEF	44.7% *	D	2.3 years	Composite of worsening HF or CV death	207	229	Reduced risk
	2064		HFpEF	44.7% *		2.3 years	Composite of worsening HF or CV death	305	381	Reduced risk
	3131		NFmrEF HFpEF	44.7% *		2.3 years	Total number of worsening SGLT2i events and cardiovascular death	815	1057	Reduced number
				44.7% *		8 months	Change from baseline of KCCQ	N/A	N/A	Improved patient-reported symptoms
				44.7% *		2.3 years	Cardiovascular death and from any cause	497	526	No difference
DELIVER [32]	NO COPD	5567	NFmrEF HFpEF	44.6%	D	8 months	Composite of worsening heart or cardiovascular death	N/A	N/A	Mild to moderate COPD is associated with worse outcomes but did not affect the beneficial effects of Dapagliflozin
	COPD	694	NFmrEF HFpEF	46.5%	D	8 months		N/A	N/A	
EMPERIAL-Preserved Trial [33]	315		HFpEF	51.1%	E	12 weeks	Change from baseline in 6MWT	N/A	N/A	Neutral effect on exercise ability
							Change from baseline in KCCQ-TSS and CHQ-SAS dyspnoea score	N/A	N/A	No effect on specific dyspnoea score
EMPEROR-Preserved trial [34]	5988		HFpEF	44.8%	D	26.2 months	Combined risk of CV death or hospitalization for HF	415	511	Reduced risk, regardless of diabetes
							Occurrence of all adjudicated hospitalizations for HF	407	541	Reduction of hospitalization for HF
							Rate of decline in the eGFR during treatment	N/A	N/A	Reduction of rate of decline
PRESERVED-HF [35]	324		HFpEF	56.6%	D	12 weeks	Change in KCCQ-CS at 12 weeks	N/A	N/A	Improved patient-reported symptoms
							Meaningful (five points or greater) change in KCCQ-CS and -OS	N/A	N/A	Magnitude of benefit higher in patients treated with SGTL2i
							Change in 6MWT distance	N/A	N/A	Improved exercise function

* Frequency of diabetes in whole sample (heart failure with mildly reduced left ventricular ejection fraction and heart failure with preserved left ventricular ejection fraction). Abbreviations: D, Dapagliflozin; E, Empagliflozin; HFrEF, heart failure with reduced left ventricular ejection fraction; NFmrEF, heart failure with mildly reduced left ventricular ejection fraction; HFpEF, heart failure with preserved left ventricular ejection fraction; heart failure, HF; CV, cardiovascular; COPD, chronic obstructive lung disease; N/A, not applicable; KCCQ-CS, Kansas City Cardiomyopathy Questionnaire Clinical Summary Score; KCCQ-OS, Kansas City Cardiomyopathy Questionnaire Overall Summary Score; 6MWT, 6-min walk test; CHQ-SAS, Chronic Heart Failure Questionnaire Self-Administered Standardized format; eGFR, estimated glomerular filtrate rat; SGLT2i, Na^+^-glucose cotransporter-2 inhibitors.

**Table 3:** Studies that tested SGLT2i in acute decompensated heart failure.

Study	*n*	% Reduced LVEF	Treatment			Follow-Up	Results
			Type	Start	Duration		
EMPULSE [36]	530	67% ^1^	Empagliflozin 10 mg/day vs. placebo	1–5 days after hospital admission	90 days	90 days	Clinical benefit (hierarchical composite of death from any cause, number of SGLT2i events, and KCCQ-SC)
EMPAG-HF [37]	59	20.7% ^2^	Empagliflozin 10 mg/day vs. placebo	Within 12 h	5 days	30 days	25% increase in cumulative urine output without affection of renal function
EMPA-REPONSE-AHF [10]	79	100% ^3^	Empagliflozin 10 mg/day vs. placebo	Within 24 h	30 days	60 days	Increased urinary output and reduced combined endpoint (worsening HF, rehospitalization for HF or death at 60 days). No effect on VAS dyspnea, diuretic response, NT-pro-BNP, or length of hospital stay.
DAPA-RESIST [38]	54	44% ^4^	Dapagliflozin 10 mg/day vs. Metalozone 5–10 mg/day	Within 24 h	5 days	90 days	Weight reduction at up to 96 h

^1^ LVEF < 40%; ^2^ LVEF < 30%; ^3^ LVEF < 50%; ^4^ LVEF < 40%. Abbreviation: LVEF, Left ventricular ejection fraction, HF heart failure; VAS, Visual Analogue Scale; KCCQ-SC, Kansas City Cardiomyopathy Questionnaire total symptom score; HF, heart failure; SGLT2i, Na^+^-glucose cotransporter-2 inhibitors.

## Data Availability

Not applicable.
